# Antimicrobial activity of laser assisted endodontic therapy in disinfecting root canals

**DOI:** 10.6026/973206300200610

**Published:** 2024-06-30

**Authors:** Shivam Patel, Harpreet Hundal, Geeta Nishad, Nityanand Shetty, Vinita Banjare, Jyotsna Sethumadhavan, Ramanpal Singh Makkad, Anushree Tiwari

**Affiliations:** 1 Private Practitioner, Monica Dental Clinic and Implant Centre, Nadiad, Gujarat, 387001, India; 2 Private Practitioner, Monga Dental Clinic, Verka, Amritsar, Punjab, 145001, India; 3 Department of Conservative Dentistry and Endodontics, Triveni Institute of Dental Sciences and Hospital, Bodri-495220, Bilaspur, Chhattisgarh, India; 4 Department of Orthodontics, Bharati Vidyapeeth (Deemed to be University) Dental College and Hospital, Navi Mumbai, Maharashtra, 400614, India; 5 Department of Dentistry, Government Medical College, Rajnandgaon, Chhattisgarh, India; 6 Department of Prosthodontics and Crown and Bridge, Bharati Vidyapeeth (Deemed to be University) Dental College and Hospital, Navi Mumbai, Maharashtra, 400614, India; 7 Department of Oral Medicine and Radiology, New Horizon Dental College and Research Institute, Bilaspur, Chhattisgarh, India; 8 Clinical Quality and Value, American Academy of Orthopaedic Surgeons, Rosemont, USA

**Keywords:** LASER, root canals, disinfection

## Abstract

Light Amplified Stimulated Emission of Radiation (LASER) is nowadays widely studied regarding their use in endodontics and restorative dentistry. Therefore, it is of interest to evaluate the antibacterial activity of three types of LASERs namely CO2 LASER.Er, Cr:YSGG LASER and Diode LASER in disinfection of root canals. 70 patients (105 single rooted teeth) were included in the study. There was application of 2% Sodium Hypochlorite (NaOCl), 2780 nm Er,Cr:YSGG LASER, 900 nm Diode LASER and CO2 LASER. Microbial samples were collected from root canals both before and after the interventions through paper points. These parameters were evaluated in microbiology laboratory to obtain Log10 Colony Forming Units (CFUs). There was significant reduction in CFUs of microorganisms inside root canal in all three LASERs evaluated and NaOCl. The reduction in CFUs in LASERs was comparable to NaOCl. Then secondly we applied each LASER in combination with NaOCl. It was observed that reduction in CFU was greater when combination of LASER with NaOCl was applied as compared when applied alone. It can be inferred that LASER when applied with NaOCl can have significant role in disinfection of root canals.

## Background:

In order to guarantee effective, enduring root canal treatment, endodontist must thoroughly clean the root canal network [1,2]. The majority of root canal treatments are performed to cure infected endodontal tissue, which can get infected indirectly through the accessory canals or apical foramina, the pulp chamber, or the dentinal tubules [3,4]. The endodontic flora then becomes mixed and mostly anaerobic. In order to adequately shape and disinfect the root canal, instruments alone is insufficient to remove germs, particles, and the smear layer; sufficient irrigation is also required[4, 5].Two percent sodium hypochlorite is the preferred irrigant at the moment. Although it has been noted that some microbes may hide and thrive within tubules or such inaccessible regions, the efficiency of hypochlorite is well established [6,7].It can be challenging to treat some pulpal disorders, particularly acute infections, and one might need to temporarily employ intracanal treatments [8, 9]. Due to issues with bacterial resistance including allergies, products like formulated or phenolated antibacterial treatments and localized antibiotics have been discontinued. Currently, the most utilized temporary intracanal therapy is calcium hydroxide [10,11]. While it works well to get rid of endodontic bacteria, hydrogen requires reapplications over an extended period of time, which can be bothersome. New methods of sterilization have been suggested in an effort to shorten the duration of therapy. In recent years, LASERs have been used more widely in healthcare, including odontostomatology [12,13]. Due to its capacity to evaporate, coagulate, reduce postoperative pain, speed up cicatrization, and incision without bleeding, the CO2 LASER has become increasingly popular in the discipline of dental surgery [14, 15, 16, 17]. Numerous studies have assessed how well LASERs sterilize root canals and have found that the amount of germs in a variety of models significantly decreased in vitro [18, 19-20]. The Erbium LASER, Er,Cr:YSGG, is a member of the infrared Erbium spectrum. It reacts with aqueous liquids and effectively eliminates hard dental tissue with intensity of 3.5 W or above [21, 22-23]. As Er,Cr:YSGG only penetrates the dentin to a thickness of 17 µm, it is employed in endodontic therapy to eliminate the layer of smear from the walls of root canal. Either rapid intracellular evaporation of water or microbial dehydration is required for it to have bactericidal effects. Er,Cr:YSGGLASER has repeatedly demonstrated better efficacy in removing smear layers than irrigation with EDTA [24, 25, 26- 27].Another of the near-infrared LASERs, the 940 nm diode LASER, is captured most by hemoglobin as well as melanin, and less by water or hydroxyapatite crystals [13-17]. Due to this characteristic, diode LASERs can effectively combat germs by penetrating deeper into dentine [18- 21]. In endodontic therapy, a 940 nm diode LASER is utilized for decontaminating root canals. By altering the structure of bacterial cells and rupturing the cell membrane, diode LASERs have a potent antibacterial impact [17-22]. The approachable bacteria are subjected to a photo-thermal action by the diode LASER. Additionally, it has a photo-disruptive effect on bacteria that are inaccessible to humans [21-24]. In these cases, cell death may not happen right away; instead, sublethal damage is caused, which inhibits the growth of the bacteria by destroying the integrity of their cell walls and accumulating denatured proteins, which stops the bacteria from growing and leads to cell lysis [22- 26]. Very tiny amounts of heat have this impact on bacteria [15- 19].There have been very few studies that have evaluated Co2 LASER. Er,Cr:YSGGLASER and Diode LASER in disinfection of root canals. Therefore, it is of interest to evaluate the antibacterial activity of three types of LASERs namely CO2 LASER. Er,Cr:YSGGLASER and Diode LASER in disinfection of root canals.

## Methods and Materials:

It was a prospective study where 70 study participants of both gender and age 18 -35 years were included.

## Inclusion criteria:

[1] Individuals with tooth having single root in anterior region of maxilla that required root canal therapy having following features

[2] Tooth having a closed apex,

[3] Tooth having a necrotic pulp,

[4] Tooth having asymptomatic apical periodontitis

[5] Individuals between the ages of 18 and 35 years.

[6] A periapical index grade of 3 or 4 (Ørstavik, *et al.*) is required for the periapical lesion to be included in this investigation.[25] On the basis of a clinical, radiological, and history-taking assessment, non-vital pulp was diagnosed.

## Exclusion criteria:

Patients with pain, swelling, fistulous tract and periodontal pockets measuring more than 3mm were excluded. Individuals with any systemic illness, allergies to NSAIDs, prior root canal therapy in the tooth in question, and recent antibiotic use were also disqualified. The vulnerable groups-pregnant women and people with physical or mental disabilities-were left out. Individuals who declined to partake in the research as well as those who encountered technical challenges-such as bent roots or fractured files-during the root canal procedure were not included in the study. Before each patient signed the informed permission form, they were fully informed about the purpose of the study and its methods.

## Distribution of study participants:

70 patients (105 teeth) were included in the study. The study participants were divided into seven categories in following manner. (Table 1)

Washing with ten millilitres of 0.125 percent chlorhexidine gluconate mouthwash for one minute was the method used to provide infection prevention of the mouth and throat. At the injection location, lidocaine topical anesthesia gel was administered. A local anesthetic solution was used for buccal infiltration in order to anesthetize the area around the tooth. Using an appropriate clamp with rubber dam, single-tooth separation was carried out. The separated tooth and rubber dam were cleaned with an antiseptic solution. Using a sterile bur, any caries as well as coronal fillings were completely eliminated without exposing the pulp chamber. With a sterilized round carbide bur with size #4, the access cavity was made ready. Utilizing a hand K-file size 15 made of stainless steel, the condition of the canals was assessed. We used one milliliter of a saline solution that was sterile to irrigate the root canal. After using an H-file to scrape the root canals, one milliliter of sterile saline solution was used as irrigation. The procedure previously reported by Gomes *et al.* [26] was modified to collect the microbial samples before intervention and evaluate the preliminary colonizers of the root canals. Three sterile paper tips were inserted into root canal with pumping movement for one minute. To prevent contamination, effort was taken to ensure that the paper points and walls of access cavity did not come into touch. They were moved right away to the microbiology department and placed into sterile tubes with a thioglycolate transport medium inside. In accordance with the instructions provided by the manufacturer, ProTaper Next nickel-titanium rotary devices were used for biomechanical preparation of root canals. In compliance with the disinfection process, the patients were split up into seven groups. Three sterile ProTaper Next absorbent paper points the same size as the master file were used to dry the canals in each group after sterile saline had been added. The final microbial sample after intervention was taken to evaluate the state of the root canal shortly before obturation and the degree of bacterial colonization after each paper point was left for one minute.

## Microbiological analysis and bacterial counts:

The tubes containing paper points placed in medium for transportation were put in a micro centrifuge then vortexes for 30 seconds after the specimens arrived at the microbiological lab. To determine the microbiological burden of commonly occurring aerobes as well as anaerobes discovered in each root canal, 100 µl aliquots of the vortexed specimens were deposited in a fresh sterilized tube bearing 1 ml of thioglycolate. Each plate's bacterial colony count was measured and expressed as colony-forming units per milliliter, or CFU/ml.

## Statistical analysis:

By examining the distribution of data and applying normalcy tests (Kolmogorov-Smirnov and Shapiro-Wilk tests), numerical data were examined for normality. A non-normal distribution was seen in the percentage reduction of the bacterial counts data. The values of the median, range, mean, and standard deviation (SD) were displayed for the data. The changes in bacterial counts within each group following disinfection were evaluated using Friedman's test. Utilizing the Kruskal-Wallis test, comparisons between categories were made. When Friedman's test or the Kruskal-Wallis test is significant, pairwise comparisons using Dunn's test are performed. A significant threshold of P ≥0.05 was established. With IBM SPSS Statistics for Windows, Version 23.0, statistical analysis was carried out. NY / Armonk: IBM Corp.

## Results:

The mean CFU at baseline in all categories was 5.36±0.07. The mean CFU values post irrigation with (NaOCl) was 0.81±0.003. There was significant decrease in colonies of microorganisms after irrigation. Similarly, mean CFU values post irradiation with CO2 LASER, Er,Cr:YSGGLASER, Diode LASER was 0.98± 0.001, 0.94±0.001 and 1.04±0.51 respectively. The mean CFU values post intervention was minimum in NaOCl group. It can be inferred that there was significant reduction in CFU after applying NaOCl, CO2 LASER, Er,Cr:YSGGLASER and Diode LASER. The reduction in CFU was comparable in each category (Table 2).

Mean CFU values post intervention in Sodium Hypochlorite (NaOCl)+CO2 LASER, Sodium Hypochlorite (NaOCl)+ Er,Cr:YSGGLASER and Sodium Hypochlorite (NaOCl)+ Diode LASER was 0.23±0.004, 0.18± 0.002 and 0.14±0.003 respectively. When NaOCl was applied along with LASER then the reduction in CFU was greater in each NaOCl+LASER as compared to NaOCl and different LASER applied independently (Table 3).

## Discussion:

Certain pulpal illnesses, especially acute infections, might be difficult to treat, and intracanal therapies may be necessary temporarily. Products like formulated or phenolated antibacterial treatments and targeted antibiotics have been abandoned due to problems with bacterial resistance, including allergies [12-19]. At the moment, calcium hydroxide is the most often used temporary intracanal treatment. Although hydrogen works well to eliminate endodontic bacteria, it must be applied again over an extended period of time, which can be inconvenient [13- 20]. There have been suggestions for new sterilization techniques aimed at reducing the length of therapy. In the field of dentistry, particularly odontostomatology, LASERs have become increasingly common in recent years [15-18].The effectiveness of Er,Cr;YSGG's smear layer removal was assessed in vitro in some studies. They came to the conclusion that Er,Cr:YSGGLASER irradiation removes the smear layer more effectively than NaOCl irrigation [12-18]. In terms of detritus and smear layer removal some researchers [8-16] also conducted an in vitro comparison between EDTA and Er,Cr:YSGGLASER. They found that the LASER was more effective at cleaning than EDTA and that using both LASER and EDTA together increased the cleanliness even more because of the cumulative effect [11-16].Findings of our study are similar to findings of some other studies. The bactericidal effects of Er,Cr:YSGGLASER irradiation independently and in combination with NaOCl irrigation against endodontic microorganisms were assessed in a study. They came to the conclusion that when combined with NaOCl irrigation, the Er,Cr:YSGGLASER has a greater capability for purification [10-19]. Owing to its ability to coagulate, evaporate, lessen discomfort following surgery, accelerate cicatrization, and make incisions without bleeding, the CO2 LASER has grown in popularity within the field of dental surgery [18- 27]. Several research evaluating the sterilization efficacy of LASERs in root canals have reported a considerable reduction in the number of germs in vitro across a range of models [20- 25].One of the elements of the infrared Erbium spectrum is the Erbium LASER, Er,Cr:YSGG. At an intensity of 3.5 W or higher, it combines with aqueous solutions to effectively remove hard tooth tissue [11- 18].

Er,Cr:YSGG is used in endodontic therapy to remove the layer of smear from the root canal walls since it only reaches a thickness of 17 µm in the dentin. It must cause either microbial dehydration or fast intracellular evaporation of water in order to exert bactericidal effects [4-7].Er,Cr:YSGGLASER has proven time and time again to be more effective than EDTA irrigation at removing smear layers [5- 12].The bactericidal capability of 2780-nm Er,Cr:YSGG and 940-nm diode LASERs was assessed in vitro by some scientists. They came to the conclusion that the wavelength combination of Er,Cr:YSGG and 940 nm diode LASER is similar to needle irrigation with NaOCl and EDTA and is safe and much more successful than each LASER alone [12- 19]. The findings are not according to our study because we observed that reduction n CFUs was greater n NaoCl + LASER as compared to NaOCl alone. It can be inferred that LASER when applied with NaOCl can have significant role in disinfection of root canals. Melanin and hemoglobin absorb most of the 940 nm diode LASER, a different type of near-infrared LASER, while water and hydroxyapatite crystals absorb less of it [9-16]. This feature allows diode LASERs to penetrate deeper into dentine, which helps them fight germs more successfully [19- 26].A diode LASER operating at 940 nm is used in endodontic therapy to clean root canals. Diode LASERs have a strong antibacterial effect by changing the structure of bacterial cells and rupturing the cell membrane. The diode LASER applies a photo-thermal action to the bacterial targets [11-16]. Applying a 940-nm diode LASER and saline, a study observed an average bacterial decrease of 98.66% in vitro [14- 21]. Furthermore, it was discovered by a study that the administration of NaOCl and EDTA along with intracanal treatment in vivo using a Diode LASER led to a 99.73% decrease in aerobic and 99.9% decrease in anaerobic bacteria [22-27].In teeth with apical periodontitis, a research examined the results of LASER operated endodontic therapy/retherapy applying a 940-nm diode LASER and EDTA vs the traditional procedure of NaOCl and EDTA. They came to the conclusion that the endodontic regimen aided by a 940-nm diode LASER is a successful substitute for traditional therapy [16-23]. These observations are similar to observations of our study. The Diode/EDTA treatment has the benefit of reducing the need for systemically administered antibiotics and chemical irrigants while also accelerating the resolution of periapical lesions [14- 20].Unfortunately, because the effectiveness of a LASER relies on both the wavelength employed and the shade of color of the area being irradiated, one type of LASER is insufficient for every purpose [14, 15]. CO2 and Nd:YAGLASERs, which both operate in the infrared region of the electromagnetic spectrum, are two distinct kinds of LASERs that have demonstrated potential in dentistry including oral surgery [16, 17].LASERs have been suggested for a number of applications in the field of restorative dentistry, including sealing pits and fissures, treating dentinal hypersensitivity, caries curettage and enamel etching. Because of its bactericidal properties, LASERs are employed in endodontics to both enlarge root canals (5) and sterilize them [17, 19].

## Conclusion:

Data shows that LASER when applied with NaoCl have significant role in disinfection of endodontic root canals.

## Figures and Tables

**Table 1:** Distribution of study participants

**Category**	**Intervention**	**Number**
Category 1	Sodium Hypochlorite (NaOCl) alone	15
Category 2	CO2 LASER alone	15
Category 3	2780 nm Er,Cr:YSGG LASER alone	15
Category 4	900 nm Diode LASER alone	15
Category 5	Sodium Hypochlorite (NaOCl)+ CO2 LASER	15
Category 6	Sodium Hypochlorite (NaOCl) + 2780 nm Er,Cr:YSGG LASER	15
Category 7	Sodium Hypochlorite (NaOCl)+ 900 nm Diode LASER	15

**Table 2:** Log10 CFU in different LASER group and sodium hypochlorite applied independently

	**Sodium Hypochlorite (NaOCl)**		**CO2 LASER**		**Er,Cr:YSGG LASER**		**Diode LASER**		**P value**
	**Pre-irrigation**	**Post irrigation**	**Pre irradiation**	**Post irradiation**	**Pre irradiation**	**Post irradiation**	**Pre irradiation**	**Post irradiation**	
Log10 CFU (Mean± SD )	5.36±0.07	0.81±0.003	5.36±0.07	0.98± 0.001	5.36±0.07	0.94±0.001	5.36±0.07	1.04±0.51	0.976
P value	0.001		0.001		0.001		0.001		

**Table 3:** Log10 CFU in different LASER group and sodium hypochlorite applied in combinations

	**Sodium Hypochlorite (NaOCl)+ CO2 LASER**		**Sodium Hypochlorite (NaOCl)+ Er,Cr:YSGG LASER**		**Sodium Hypochlorite (NaOCl)+ Diode LASER**		**P value**
	**Pre-intervention**	**Post intervention**	**Pre intervention**	**Post intervention**	**Pre intervention**	**Post intervention**	
Log10 CFU (Mean± SD )	5.36±0.07	0.23±0.004	5.36±0.07	0.18± 0.002	5.36±0.07	0.14±0.003	0.654
P value	0.001		0.001		0.001		
